# Separation of *Pseudomonas aeruginosa* type IV pilus-dependent twitching motility and surface-sensing responses

**DOI:** 10.1128/mbio.02521-25

**Published:** 2025-10-07

**Authors:** Rebecca Barnshaw, Hanjeong Harvey, Matthew McCallum, Tomas Lazarou, Sheryl Nguyen, Ikram Qaderi, Veronica Tran, Nathan Roberge, Christopher Geiger, George A. O'Toole, P. Lynne Howell, Lori L. Burrows

**Affiliations:** 1 Department of Biochemistry and Biomedical Sciences, McMaster University 3710, Hamilton, Canada; 2 Michael G. DeGroote Institute for Infectious Disease Research, McMaster University, Hamilton, Canada; 3 Department of Biochemistry, University of Toronto 233836, Toronto, Canada; 4 Program in Molecular Medicine, The Hospital for Sick Children 7979, Toronto, Ontario, Canada; 5 Department of Microbiology and Immunology, Geisel School of Medicine at Dartmouth 12285, Hanover, New Hampshire, USA; University of California, Berkeley, Berkeley, California, USA

**Keywords:** cell motility, type IV pili, *Pseudomonas aeruginosa*, cyclic AMP, ATPase

## Abstract

Many functions of *Pseudomonas aeruginosa* type IVa pili, including twitching motility and surface sensing, depend on dynamic cycles of filament assembly and disassembly powered by the cytoplasmic ATPases PilB, PilT, and PilU. Deletion of *pilT* results in loss of twitching and pilus-specific bacteriophage susceptibility, while non-twitching *pilU* mutants remain susceptible to pilus-specific phages, indicating that they still produce retractable pili. *pilU* mutants have high basal levels of the secondary messenger cyclic AMP (cAMP) that normally increases following surface contact, suggesting aberrant surface sensing. To better understand PilU’s role in pilus biology, we solved its X-ray crystal structure and used phylogenetic analyses to identify conserved differences between PilT and PilU. Chemical mutagenesis followed by whole-genome sequencing was used to identify suppressors in the Δ*pilU* mutant background that restored twitching motility. The mutations mapped to the major pilin, PilA, or the pilus tip adhesin, PilY1. Both the position and nature of the substitutions in PilA impacted restoration of motility, and it was dependent on functional PilT. Complementation of most *pilU* suppressors with PilU *in trans* further increased motility, while the expression of wild-type PilA *in trans* decreased motility in a dose-dependent manner. Notably, cAMP levels remained elevated in most twitching *pilU* suppressor mutants, showing that surface sensing and motility can be uncoupled. Together, our data suggest that the bacterial response to surfaces reflects a complex interaction of PilU function with specific alleles of PilY1 and PilA that together modulate pilus dynamics and function.

**IMPORTANCE:**

The ability of bacteria to sense and respond to contact with surfaces is important for triggering changes in secondary messenger levels and gene expression, leading to the formation of biofilms and increased production of virulence factors. For *Pseudomonas aeruginosa*, the expression of functional type IVa pili is important for the accumulation of cyclic AMP (cAMP) following surface contact. Deletion of the PilT retraction ATPase paralog PilU leads to loss of pilus-mediated twitching motility but also high intracellular levels of cAMP, a phenotype mimicking that of surface-adapted cells. Here, we isolated twitching suppressors of a *pilU* deletion mutant that mapped to the pilin subunit PilA or pilus-tip adhesin PilY1 and showed that for most, elevated cAMP levels did not decrease when motility was restored. Twitching was dependent on functional PilT, and complementation with PilU further increased twitching for most mutants. These data show that in permissive contexts, PilU is not required for twitching motility, providing new insights into mechanisms of bacterial surface sensing and evolution of type IVa pilus motor function.

## INTRODUCTION

Type IV pili (T4P) are common prokaryotic surface appendages belonging to the ancient and functionally diverse type IV filament family (1). These dynamic fibers are repeatedly and rapidly assembled and disassembled by dedicated nanomachines embedded in the cell envelope. T4P are involved in multiple functions, including adherence to and sensing of surfaces, twitching motility, DNA uptake, biofilm development, and bacteriophage (phage) predation, and they are important virulence factors (2–5). There are three major subfamilies—T4aP, T4bP, and T4cP (Tad)—with signature differences in specific components of the machineries, but all share common elements (1, 6). In general, pilin subunits interact with a cytoplasmic membrane-embedded platform protein, whose cytoplasmic domains interface with one or more hexameric ATPases (7). Conformational changes in the ATPases resulting from coordinated cycles of ATP hydrolysis around the hexamer are thought to cause rotation and translation of the platform protein, inserting associated pilin subunits from the cytoplasmic membrane into a helical filament during extension or vice versa during retraction (8, 9). How the machinery switches between extension and retraction states remains unclear (10), as does the mechanism by which forces acting on surface-adhered filaments that are being retracted might transduce information that can influence bacterial behavioral responses (11–15).

T4aP pili are composed mainly of major PilA subunits, plus a priming subcomplex composed of five minor pilins and the non-pilin protein PilY1 that forms the pilus tip (16, 17). In the opportunistic pathogen *Pseudomonas aeruginosa*, PilY1 is a ~125 kDa protein proposed to act as an adhesin, a mechanosensor involved in surface sensing and biofilm development, and as a plug that prevents complete retraction of the pilus filament into the cell (18–22). Its role as a mechanosensor relies on the presence of a von Willebrand A (vWA)-like motif in its N-terminal domain. PilY1 is important for increased production of the second messenger cyclic-diguanylate-monophosphate (c-di-GMP) that promotes production of exopolysaccharides (EPS) following surface encounters. This regulatory output is thought to require force-induced unraveling of the vWA domain upon surface contact (18), but the exact mechanism of signal transduction from outside to inside is not yet fully understood (23).

**Fig 1:**
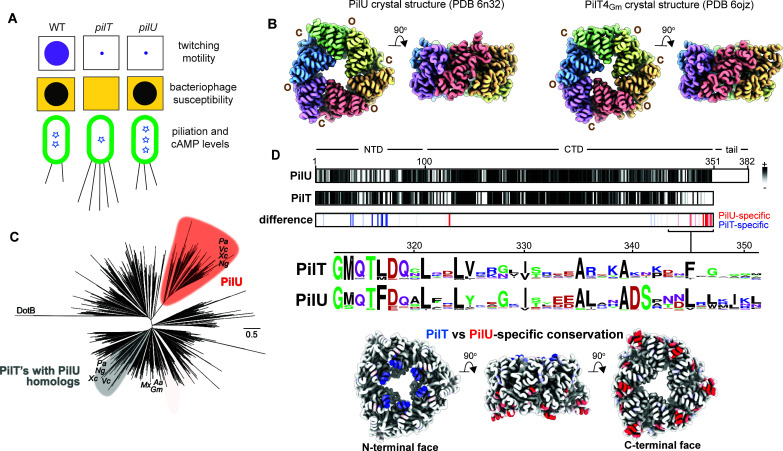
PilU is a hexameric motor ATPase with unique features. (**A**) Summary of *pilT* vs *pilU* phenotypes. While they produce surface-exposed pili, neither *pilT* nor *pilU* mutants can twitch (purple circles). *pilU* mutants remain susceptible to lysis by pilus-specific phages (black circles) and have high basal levels of cyclic AMP (cAMP) compared to wild type (stars). (**B**) Orthogonal views of the X-ray crystal structures of the PilU hexamer (left) and, for comparison, the PilT4Gm hexamer (right) are shown, both adopting the C3-symmetric OCOCOC conformation. Packing units (comprising the N-terminal and C-terminal domains of adjacent chains) are colored red, orange, yellow, green, blue, and purple. (**C**) A neighbor-joining phylogenetic tree illustrates the relationships among PilT-like ATPases most closely related to PilT and PilU. Sequences from representative model organisms are labeled, and only branches with >85% bootstrap support (1,000 replicates) are shown. DotB serves as the outgroup. Pa, *P. aeruginosa*; Ng, *Neisseria gonorrhoeae*; Xc, *Xanthamonas campestris*; Vc, *Vibrio cholerae*; Mx, *Myxococcus xanthus*; Aa, *Aquifex aeolicus*; Gm, *Geobacter metallireducens*. (**D**) Conservation analyses using species-paired PilT and PilU sequences. (Top) Residue conservation, calculated with ConSurf, is depicted in grayscale (white: low; black: high) for both PilU and PilT. Subtracting PilT conservation scores from PilU reveals residues preferentially conserved in either protein. The top 15% of PilU-enriched conserved residues are shown in red; the top 15% of PilT-enriched residues are shown in blue. *Middle*: A sequence logo for residues 313–351 (PilU numbering) highlights sequence divergence in this region with greater PilU sequence conservation. (Bottom) Orthogonal views of the PilU structure showing the conserved sets of residues (PilU-specific, red; PilT-specific, blue) mapped onto the structure.

Addition of pilin subunits from their reservoir in the cytoplasmic membrane to the growing filament is powered by the extension ATPase PilB via the platform protein PilC, while extraction of subunits from assembled filaments back into the membrane during retraction requires the ATPase PilT. *P. aeruginosa* encodes a second retraction ATPase, PilU, whose function is not clearly defined (24–27). While *P. aeruginosa pilT* mutants are hyperpiliated, incapable of twitching motility, and resistant to pilus-specific phage infection, *pilU* mutants have approximately wild-type levels of pili and remain phage susceptible, even though they do not twitch (Fig. 1A) (28). These data suggest that *pilU* mutants express retractable pili. Single-cell imaging using iSCAT microscopy confirmed that *pilU* mutants grown in liquid retract their pili in a PilT-dependent manner, suggesting that while not essential for filament disassembly, PilU may be needed to generate additional force when pili are tethered to a surface and thus under higher loads (27). Supporting this idea, recent optical tweezer experiments showed that pilus retraction stall forces in a *pilU* mutant were significantly reduced compared to wild type (26). Protein-protein interaction and purification data suggest that PilT and PilU interact but do not form mixed hexamers (29, 30). Instead, Alphafold modeling suggests that they may form stacked hexamers (31), although the orientation and stoichiometry of such complexes require experimental validation.

Levels of the secondary messenger cyclic AMP (cAMP) are positively correlated with surface adhesion and biofilm initiation in *P. aeruginosa*, phenotypes regulated by the cAMP-binding transcriptional regulator, Vfr (virulence factor regulator) (14, 32, 33). Surface attachment leads to increased T4P expression and thus increased cAMP production in a feed-forward manner (25, 34). In *P. aeruginosa* PA14, cAMP levels increase over multiple generations during reversible attachment in the initial stages of biofilm formation (<20 h after surface contact) due to repeated activation of the Pil-Chp regulatory system that controls adenylate cyclase activity (25). Following the initial contact event, each subsequent generation has higher cAMP levels than the one before. This multigenerational memory leads to an eventual sudden increase in the number of surface-adhered cells. Interestingly, PA14 *pilU* mutants have significantly higher basal levels of cAMP than wild type and take less time to establish a surface-attached population (25). In contrast, *pilA*, *pilT*, and *pilJ* (lacking the Pil-Chp chemoreceptor) mutants have lower cAMP levels and take longer to establish a population on the surface. These data suggest that PilU has a distinct role in surface sensing compared to PilT. Whether PAO1 *pilU* mutants behave similar to PA14 *pilU* mutants with respect to surface sensing has not been established.

In this study, our X-ray crystallographic analysis of PilU confirmed the expected structural similarity between PilU and PilT hexamers, while phylogenetic analyses showed key divergences in their C-terminal residues. We hypothesized that the decrease in retraction forces caused by the absence of PilU might mimic the effects of stalling of pilus retraction upon attachment to a surface, leading to increases in cAMP levels, and that restoration of motility in the *pilU* background would reduce cAMP levels to wild type. We confirmed that, like PA14, PAO1 *pilU* mutants have high basal levels of cAMP. We isolated twitching suppressor mutants in a *pilU* background that mapped to PilA or PilY1, showing that *pilU* is not necessary for motility in *P. aeruginosa*. Several *pilU* suppressors retained a high cAMP phenotype even though they could twitch, suggesting that motility and surface sensing can be uncoupled.

## RESULTS

### PilU is structurally and conformationally similar to PilT

Although PilT and PilU are paralogs essential for bacterial twitching motility, mutants lacking each of the proteins exhibit strikingly different phenotypes (Fig. 1A). To uncover the unique role of *P. aeruginosa* PilU, we first determined its crystal structure. The protein was purified as described in Materials and Methods, and the final buffer for crystallization contained 75 mM NaCl, 25 mM HEPES (pH 7.0), 125 mM (NH_4_)_2_SO_4_, 20 mM citric acid, 10% glycerol, 12.5 µM ATP, 11.5% PEG 8000, 50 mM Tris (pH 9.5), and 0.4 M LiCl. The structure was determined at 4.5 Å resolution, with four protomers in the asymmetric unit (Fig. S1). While the overall domain organization and distinctive *C*3 symmetric hexameric architecture were clearly resolved, individual side chains could not be confidently modeled and were therefore not included (Table S1).

Based on our previous definitions of packing unit interfaces formed by adjacent protomers, the PilU *C*3 hexamer has alternating open (O) and closed (C) subunits, mirroring the *C*3-symmetric OCOCOC conformation of *Geobacter metallireducens* PilT (35) (Fig. 1B). The OCOCOC conformation of PilU has been identified in PilT structures (35, 36), while the assembly ATPase PilB adopts a C2-symmetric CCOCCO conformation (8, 9). PilB has two N-terminal domains (N1D and N2D) that interact with regulatory ligands (37), while PilT and PilU have only a single N-terminal domain.

Phylogenetic analysis of PilT and PilU homologs across bacteria using a database that minimizes biases due to redundancy (31) identified three major clusters: PilT, PilU, and DotB, a distant relative associated with type IV secretion systems (Fig. 1C). Within the PilT cluster, species encoding PilU formed a distinct subcluster, suggesting co-evolution of these ATPases. Sequence comparisons between PilU and species-matched PilT homologs revealed a striking divergence within the final 60 residues of the C-terminus (Fig. 1D). While the first 30 of these C-terminal PilU residues remain structurally conserved, the last 30—typically absent in PilT—are predicted to be disordered by ESpritz (38) and were unresolved in the crystal structure. Structural predictions (39) suggest that these residues extend outward rather than integrating into the hexameric core, and recent Alphafold modeling studies suggested that this segment of PilU may stabilize its interaction with PilT (31). Together, these findings establish PilU as a co-evolved motor ATPase, likely leveraging its unique C-terminal features for distinct functional roles.

### Identification of motile suppressor mutants in a Δ*pilU* background

**Fig 2:**
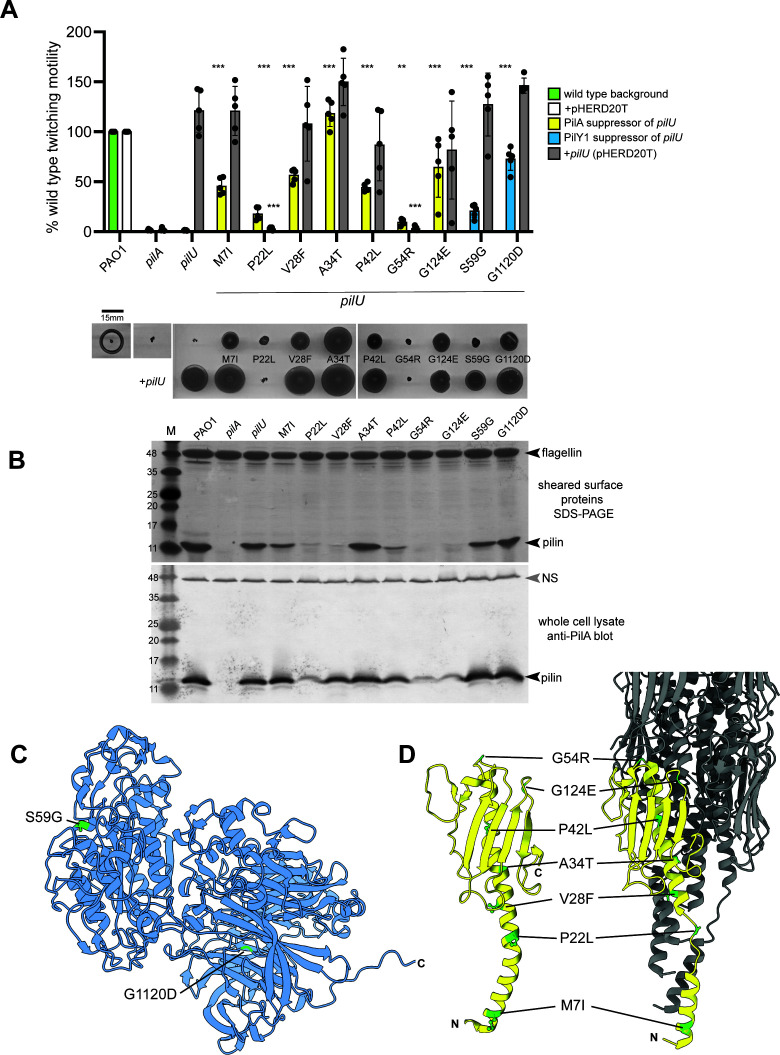
Suppressor mutations restore twitching motility in a *pilU* background. (**A**) Twitching motility of *pilU* suppressor mutants complemented with empty vector (pHERD20T) or *pilU* in *trans*. Suppressors mapping to PilA are shown in yellow, while those mapping to PilY1 are shown in blue. Representative twitching zones are shown below the graph. (**B**) Representative Coomassie blue-stained SDS-PAGE of sheared surface proteins (top) and western blot of corresponding whole-cell lysates probed with anti-PilA antisera (bottom). The flagellin subunit is used as a loading control for the gel, while a non-specific band (NS) is used for the blot. (**C**) Alphafold2 model of PAO1 PilY1 in blue, with the locations of suppressor mutations shown in green. (**D**) Alphafold2 model of PAO1 PilA monomer and filament (PDB 9EWX) forms in yellow, with the locations of suppressor mutations shown in green (numbering based on mature pilin sequence).

To further establish PilU’s functional role compared to PilT, we took a genetic approach, capitalizing on the fact that Δ*pilU* mutants, while non-motile in standard twitching assays, still produce retractable pili. We hypothesized that we could identify suppressors that regained twitching motility in standard 1% Luria-Bertani (LB) agar stab assays (40), and to increase the chance of finding such mutants, we first treated the cells with the mutagen ethyl methanesulfonate (EMS) as described in Materials and Methods. Suppressors that migrated away from the point of inoculation were selected and restreaked for single colonies, and those with reproducible twitching phenotypes were subjected to further analyses. Whole-genome sequencing and comparison using breseq (41) were used to identify relevant mutations. We isolated single-point mutations that mapped to *pilY1*, encoding the pilus tip adhesin, or *pilA*, encoding the major pilin. Each point mutation was regenerated in a clean PAO1 *pilU* background and its twitching phenotypes quantified (Fig. 2A).

Twitching requires the expression of correctly folded and processed pilins (42) and their productive assembly and disassembly by the T4P machinery. Despite its inability to twitch, a Δ*pilU* mutant has approximately wild-type levels of surface pili (Fig. 2B). A subset of the suppressors had low levels of recoverable surface pili (P22L, V28F, P42L, G54R, and G124E), but of those, only P22L, G54R, and G124E showed decreased levels of pilins in whole cells. This suggests that the V28F and P42L mutations might alter assembly-disassembly dynamics, leading to shorter (which are harder to shear) or fewer surface pili. Furthermore, the levels of surface pili recovered did not necessarily correlate with the extent of twitching motility observed.

PilY1 is a large ~125 kDa non-pilin protein predicted to have a globular N-terminal domain connected by a flexible linker to a C-terminal eight-bladed beta propeller domain (Fig. 2C) (19, 43). With minor pilins FimU, PilVWX, and PilE, it forms a tip subcomplex that primes pilus assembly and is involved in surface sensing (14, 16, 18, 22). Two gain-of-function mutations in PilY1 were identified, one in each of its two domains. S59G mapped to the N-terminus of PilY1, in the predicted vWA-like region (residues 48–368) (18), while G1120D mapped to the C-terminal domain. These mutations restored twitching motility to ~21% and 73%, respectively, of wild type.

The remaining mutations mapped to PilA, the major pilin subunit. PilA is a small lollipop-shaped protein with an extended S-shaped N-terminal α1 helix connected by an α-β loop to a flat four-stranded β-sheet terminating in a disulfide-bonded loop (Fig. 2D) (44). The α1 helix is divided into α1N, which is membrane-embedded prior to filament assembly, and α1C, which packs against the back of the four-stranded β-sheet. Type IV pilins are expressed as prepilins with a positively charged six-residue signal sequence that must be removed by the prepilin peptidase PilD prior to assembly (42, 45). The extent to which motility was restored in the *pilU* background depended on the position and properties (below) of the mutation. G54R (mature pilin numbering, after removal of the leader sequence), located in the α-β loop at the top of the subunit, only marginally increased twitching (~10% of wild type), while G124E, located in the loop between β3 and β4, increased twitching to ~60% of wild type. All other point mutations were mapped to the α1 helix. A34T motility was similar to wild type, while the remaining mutations restored intermediate levels of twitching. Complementation of the mutants with *pilU* in *trans* further increased motility, except for the P22A and G54R mutants, where motility was lost in the complemented strains (Fig. 2A). These data show that, given the appropriate context, PilU is not required for twitching motility.

### Complementation of *pilU* suppressors with wild-type PilA reduces motility

**Fig 3:**
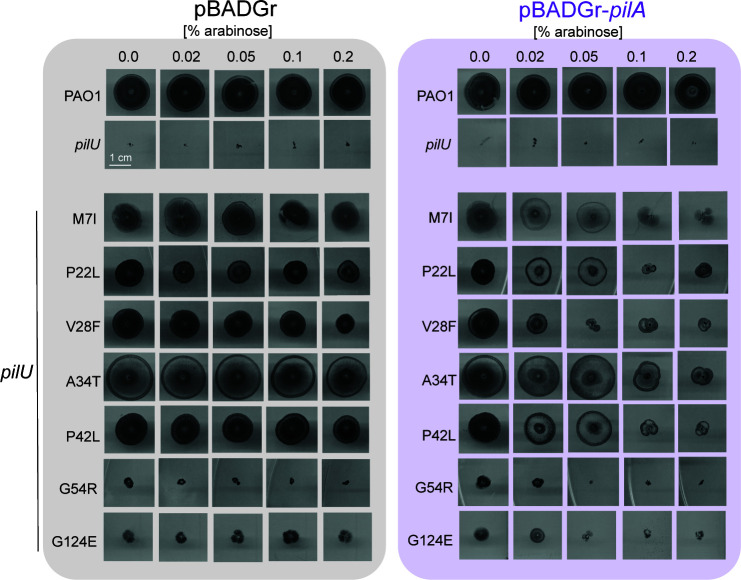
Twitching of PilA suppressors complemented with wild-type PilA. Suppressor mutants with mutations in PilA were complemented with empty vector (pBADGr, left) or pBADGr encoding wild-type PilA (right), and its expression was induced with increasing amounts of L-arabinose (% [wt/vol]).

We predicted that in strains where suppressors of Δ*pilU* mapped to *pilA*, expression of wild-type PilA *in trans* would have a dominant-negative effect on motility due to the formation of mixed filaments. We transformed each strain with the arabinose-inducible empty vector pBADGr or a construct encoding wild-type PilA and examined twitching over a range of inducer concentrations. There was a concentration-dependent decrease in motility as the amount of arabinose increased, although the magnitude of the impact depended on the individual suppressor background (Fig. 3; Fig. S2).

### A34 is a functionally promiscuous, context-dependent position in PilA

**Fig 4:**
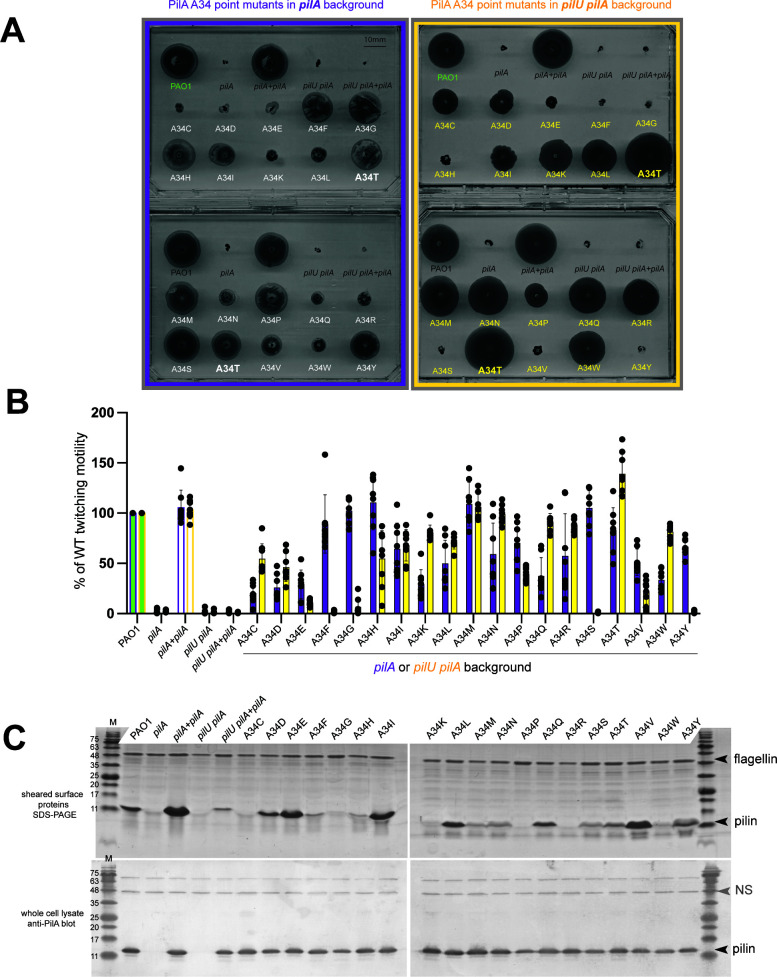
Effect of point mutations at PilA A34 on pilus function in the *pilU* background. (**A**) Representative twitching motility assays for PilA point mutants expressed in the *pilA* (purple) or *pilU pilA* (yellow) backgrounds. The original A34T suppressor (bold text) is included on all four plates for reference. (**B**) Quantification of twitching motility in the *pilA* (purple) and *pilU pilA* (yellow) backgrounds, *n* = 6. The controls for each set of plates are shown as green (wild type) or white (*pilA*) bars with purple or yellow outlines. (**C**) Representative Coomassie blue-stained SDS-PAGE (top) of sheared surface proteins from the complemented *pilU pilA* mutants and western blot (bottom) of the corresponding whole-cell lysates using anti-PilA antisera. The flagellin is used as a loading control for gels and a non-specific band (NS) for blots.

To ask whether the chemistry, as well as the position, of the point mutations in PilA is important for motility in the absence of *pilU*, we chose the A34T suppressor whose motility is similar to wild type for further characterization. We generated a set of PilA constructs with all possible amino acid substitutions at A34 and tested them for their ability to support twitching motility in the *pilA* (Fig. 4A) or *pilU pilA* backgrounds (Fig. 4B). As expected, complementation of the *pilU pilA* double mutant with wild-type *pilA* failed to restore twitching, recapitulating the phenotype of a *pilU* mutant. Interestingly, twitching motility assays revealed that all 19 substitutions at A34 in PilA supported at least some motility in the *pilA* background, while only 15 of 19 supported motility in the *pilU pilA* background. In several cases, motility in one background was opposite that in the other; one striking example was A34S, where motility was similar to wild type in the *pilA* background but abolished in the *pilU pilA* mutant. This result was remarkable, given that the original suppressor was A34T, which has a polar side chain similar to A34S, differing in length by only a single methylene, but restores wild-type levels of motility in both the *pilA* and *pilA pilU* backgrounds. There were additional cases, such as A34M, where twitching was similar to wild type, regardless of whether PilU was present. These results show that subtle changes in PilA sequence can have pronounced impacts on T4aP function, and the presence or absence of PilU can be relevant to whether a particular pilin is functional in terms of motility.

Based on whole-cell western blots, all PilA A34 point mutants were stably expressed at levels similar to wild type in the *pilU pilA* background; however, the amount of assembled surface pili that could be recovered by shearing varied (Fig. 4C). Furthermore, the amount of surface pili recovered did not necessarily correlate with motility. For example, A34E expression resulted in high levels of surface piliation but poor motility (characteristic of retraction defects), while A34R had the opposite phenotype, near wild-type motility in the *pilA pilU* background but few recoverable surface pili. These side chains have opposite charges and likely impact assembly-disassembly dynamics. A34F or A34Y substitutions resulted in the loss of motility even though pili could still be recovered, while A34W had fewer recoverable pili but near wild-type motility. Finally, the A34S transformant had wild-type levels of surface pili despite the lack of motility in the *pilU pilA* background, phenocopying wild-type PilA. These data suggest that PilA tolerates a variety of substitutions at some positions, but specific impacts on pilus assembly and motility can be hard to predict *a priori*.

### Twitching in suppressor mutants is PilT dependent

**Fig 5:**
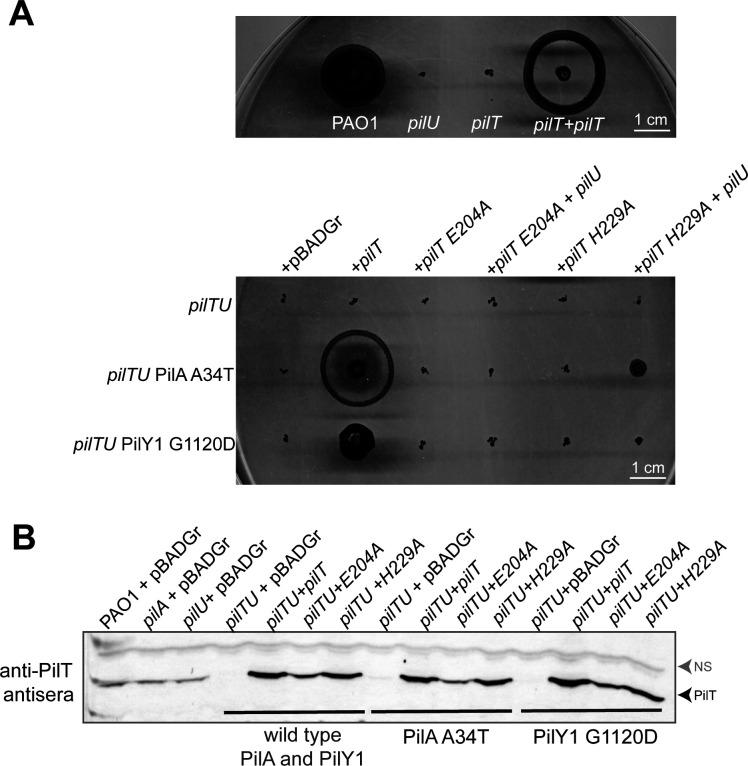
PilT function is required for the motility of *pilU* suppressor mutants. (**A**) *pilT* was deleted in the *pilU* mutant and each of the *pilU* PilA A34T and *pilU* PilY1 G1120D suppressors. Each strain was complemented with vector (pBADGr) or constructs expressing wild-type PilT or PilT mutants E204A (Walker B) or H229A (His Box). A second plasmid (pHERD20T) expressing *pilU* was added where indicated. (**B**) Western blot with anti-PilT antisera, showing that the inactive variants of PilT are expressed at levels similar to wild type. NS = non-specific band used as a loading control.

Some type IV pilus (and related type II secretion) systems lack retraction ATPases entirely, and filament retraction is proposed to result from the activity of a single bifunctional ATPase, or from spontaneous disassembly of subunits (1, 46–51). In other cases, PilU can substitute for PilT function, if a non-functional PilT is still present to connect PilU to the assembly system (29, 30). To determine if twitching of our *pilU* suppressors was dependent on having at least one functional retraction ATPase, we used the PilA A34T suppressor that had wild-type levels of motility, as well as the PilY1 G1120D suppressor that had intermediate motility. When we deleted *pilT*, the strains no longer twitched (Fig. 5). When we complemented the *pilT pilU* suppressor triple mutants with wild-type PilT or an inactive PilT variant with a point mutation in the Walker B motif that coordinates Mg^2+^ for ATP hydrolysis (52), only wild-type PilT restored motility, confirming that twitching of the suppressors was dependent on functional PilT. Finally, we complemented the *pilT pilU* suppressor triple mutants with both PilU and the inactive Walker B mutant of PilT to link PilU to the T4aP machinery, but motility was not restored (Fig. 5). These data show that motility requires a functional copy of PilT even if PilU is present. When the same experiments were performed with an H229A His Box mutant of PilT (53), we saw a small amount of twitching only in the background where both PilU and PilT H229A were expressed with PilA A34T, consistent with previous reports that this version of PilT supports phage infection and is thus partly functional (35). However, these results confirm that PilU augments PilT function, as PilT H229A alone could not support motility in the A34T background when PilU was absent.

### Twitching motility of *pilU* suppressors does not correlate with cAMP levels

Surface sensing by *P. aeruginosa* T4aP leads to increased production of cAMP and expression of the extensive Vfr regulon (14, 25, 32, 54). Mutants lacking *pilU* have elevated levels of cAMP compared to the wild type, accelerating their irreversible colonization of surfaces. Kuchma and O’Toole (55) showed that pilus assembly was necessary but not sufficient for signaling, and motor configurations that supported phage infection but not twitching (such as that of a *pilU* mutant) still permitted signaling. Using our suppressor mutants, we asked whether restoration of twitching motility in the *pilU* background could decrease its elevated cAMP levels.

**Fig 6:**
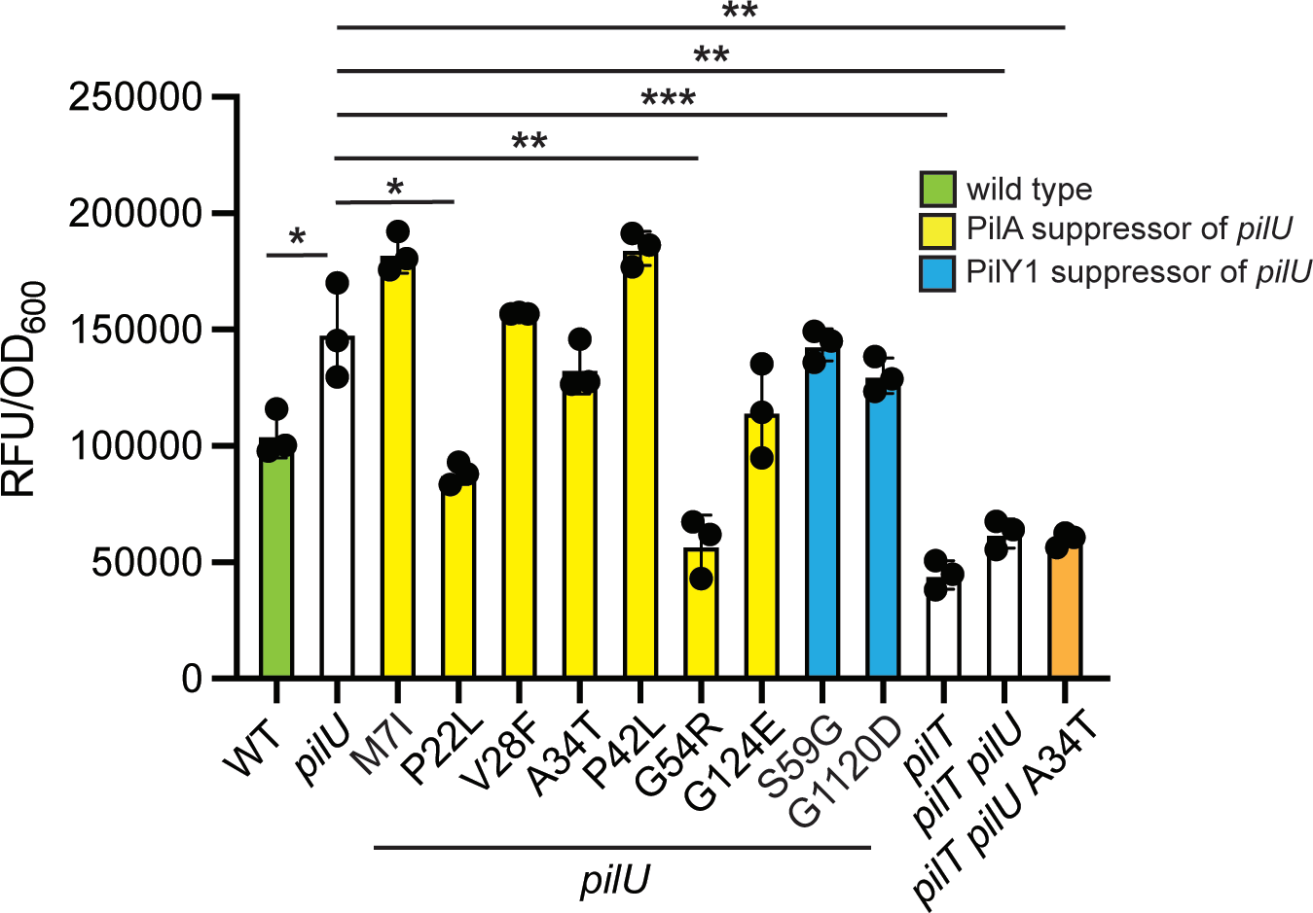
cAMP levels in *pilU* remain elevated in most suppressor mutants. cAMP levels were estimated using a fluorescent reporter system as described in Materials and Methods. Most *pilU* suppressor mutants retained the high cAMP phenotype of the parent strain despite their gain in twitching motility. Loss of *pilT* resulted in significantly decreased cAMP relative to *pilU* in the *pilU* or *pilU* PilA A34T (orange bar) backgrounds. *, *P* < 0.05; **, *P* < 0.001; ***, *P* < 0.0001.

Each of the strains of interest was transformed with a cAMP-responsive transcriptional reporter (14, 54) to estimate cellular cAMP levels (Fig. 6). We confirmed that deletion of *pilU* causes a significant increase in cAMP levels compared to the wild type. Interestingly, restoration of twitching motility in the *pilU* background did not necessarily correlate with decreased levels of cAMP. For example, A34T restores wild-type levels of motility, but its cAMP levels were not significantly different from the Δ*pilU* parent. Its high cAMP levels were dependent on the expression of functional pili, as loss of *pilT* in both Δ*pilU* and the A34T suppressor decreased reporter activity to levels similar to the *pilT* control. Other suppressors, such as P22L and G54R, restored only limited motility (Fig. 2A) but showed significant decreases in cAMP compared to Δ*pilU*. These data suggest that motility can be uncoupled from the high cAMP levels in the Δ*pilU* background, and PilA sequence can influence cAMP levels.

### *pilU* suppressors impact other surface-associated phenotypes

**Fig 7:**
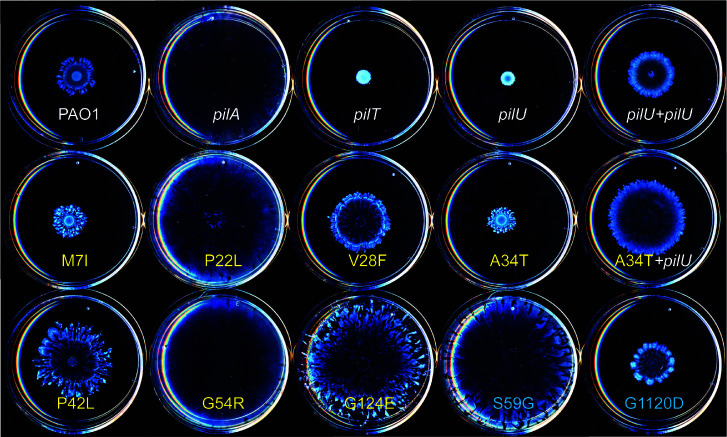
Swarming motility of *pilU* suppressor mutants. Representative swarming images for *pilU* suppressor mutants and controls on Davis minimal-glucose-casamino acid-agarose (DmGCA) plates. PilA suppressors of *pilU* are labeled in yellow text, and PilY1 suppressors are labeled in blue. The *pilA*, P22L, G54R, and S59G strains swarmed to the edges of the plate over the 6 h incubation period. For scale, each dish is 5 cm in diameter.

The cumulative increases in cAMP resulting from successive rounds of surface contact enhance cellular attachment and establishment of biofilms (25). High levels of cAMP in Δ*pilU* mutants compared to wild type accelerated their irreversible attachment, suggesting that they behave like cells that have already been in contact with a surface. Since many of the suppressors we isolated retained high levels of cAMP despite their increased twitching motility, we decided to examine other surface-associated behaviors, specifically biofilm formation (Fig. S3) and swarming motility (Fig. 7). In our batch culture assay format, biofilm formation by *pilA* or Δ*pilU* mutants was not significantly different from wild type, and most suppressors were not significantly different from the Δ*pilU* parent strain. However, the two *pilU* suppressors that restored the least twitching motility, P22L and G54R (Fig. 2A), made significantly less and significantly more biofilm, respectively, than their Δ*pilU* parent. While these two suppressors have among the lowest cAMP levels (Fig. 6), other mutants with low cAMP levels, such as *pilT*, made wild-type levels of biofilm. These data suggest that the amount of pili expressed on the cell surface and the level of cAMP in the cells have a limited impact on biofilm formation in this format, but specific pilin mutations might have regulatory consequences that should be further explored.

On DmGCA swarming plates, the non-piliated *pilA* mutant was a hyperswarmer compared to wild type, while the non-twitching but piliated *pilT* and *pilU* strains were unable to swarm. Complementation of Δ*pilU* with *pilU* in *trans* restored wild-type levels of swarming. The phenotypes of these controls imply that expression of pili that do not support twitching motility interferes with swarming, while the lack of surface pili allows for hyperswarming. P22L and G54R, which have limited twitching motility and few surface pili (Fig. 2A), resembled a *pilA* mutant, swarming over the entire plate. G124E, which twitches ~60% of wild type but has very few recoverable surface pili, was also a hyperswarmer. In contrast, A34T, which twitches similarly to the wild type, had the least swarming motility compared to the others. We expected that complementation of A34T with *pilU*—which further increases its twitching (Fig. 2A)—might decrease swarming, but unexpectedly, its swarming increased. Interestingly, the PilY1 S59G suppressor, which restores only ~21% of wild-type twitching, is a hyperswarmer even though it has wild-type levels of intracellular PilA and expresses surface pili, similar to its PilU parent (Fig. 2B). Previous studies showed that PilY1 regulates swarming and virulence independently of its role in pilus assembly, and deletion of *pilY1* increases swarming (22). From these data, we conclude that—with the exception of the PilY1 point mutant S59G—strains with low levels of surface pili swarm well; strains that can twitch well can swarm regardless of whether they express PilU; and strains that express pili but cannot twitch (*pilT* and *pilU*) cannot swarm in the absence of second-site mutations that restore twitching.

## DISCUSSION

Our X-ray crystal structure of PilU showed a typical PilT-like hexameric ATPase architecture, captured in a *C*3 OCOCOC configuration (35). These data support the conclusion that it is a motor protein that assists PilT in pilus retraction under high loads, a hypothesis supported by recent studies showing that while *pilU* mutants can retract their pili, they generate reduced retraction forces compared to wild-type cells (26, 29, 30, 56). This is also consistent with the inability of *pilU* mutants to twitch in standard sub-surface assays (24), a high-friction environment. Prior phylogenetic analyses of Pseudomonads (31) plus the broader analyses performed here showed that PilU homologs form a unique subgroup within the PilT-like family. In species with PilU homologs, PilT homologs are more similar to one another than to stand-alone PilTs, implying their co-evolution. Structural modeling, two-hybrid studies, and functional assays suggest that PilT and PilU interact directly to generate increased retraction forces under high loads, although the details of their stoichiometry and orientation relative to one another and the pilus machinery remain to be clarified (29–31, 57–59). Some structural predictions suggest that PilT and PilU hexamers might interact in a back-to-back manner via their C-terminal faces, and the C-terminal residues of PilU that are unstructured in the crystal might wrap around PilT to stabilize their interaction (31).

In a Δ*pilU* background where PilT is the only retraction ATPase, single residue changes in PilA or PilY1 sequence were sufficient to restore twitching, from barely (G54R) to essentially wild-type levels (A34T). Therefore, given the right context, PilT alone is capable of generating sufficient force to overcome friction between the cell body and the surface. This finding may explain why many species have only PilT homologs; their pilins and/or adhesins may have “permissive” sequences. As exemplified by our A34S and A34T variants of PilA, the shift from a non-permissive to permissive pilin sequence can be as subtle as a conservative Ser to Thr substitution. As a corollary, the current wild-type PAO1 PilA sequences may have evolved to optimize strong interactions between subunits—or, in the case of PilY1, with surfaces—to support vigorous motility, creating a scenario in which forces provided by PilT alone were no longer sufficient for pilus function. This may have led to the selection of a *pilT* gene duplication event and subsequent adaptation of PilU to bolster PilT function. This idea could also explain why some bacteria, including *Neisseria* and *Geobacter*, have accumulated three or four retraction ATPase homologs (59–62). In *Neisseria*, PilT, PilT2, and PilU interact (59), and PilT2 doubles the speed of pilus retraction and motility (63), suggesting that it plays a supporting role similar to PilU in *P. aeruginosa*.

Studies of the type IVa competence (Com) pili in *Vibrio cholerae* showed that loss of PilU had minimal impact on transformation efficiency, likely because DNA uptake requires lower retraction forces compared to motility (64, 65). In enzymatically inactive PilT Walker A or Walker B mutants, transformation became dependent on PilU, while deletion of *pilT* led to the loss of transformation even in the presence of PilU (29, 30). Those data imply that PilT, whether functional or not, connects PilU to the pilus machinery. In *P. aeruginosa*, *pilU* mutants remain phage susceptible, showing that PilT supports limited pilus retraction. Deletion of PilT leads to phage resistance, but in strain PA14, its inactivation via point mutation in the Walker A motif allows for phage infection if PilU is present (66). Our data in strain PAO1 (Fig. 5) confirmed that PilU can support limited motility of a PilT His Box mutant but only if a permissive PilA allele such as A34T is also present. Together, these data support the consensus that PilU cannot act as an independent motor unless PilT connects it to the machinery. Alternatively, PilU might regulate PilT activity. This idea has precedent in studies of the assembly ATPase PilB, where multiple factors ranging from small molecules (Zn^2+^, cyclic-di-GMP) to proteins (PilZ and FimX) can modulate its activity (37).

How do single-residue changes in PilA or PilY1 make them permissive for motility in a *pilU* background? Point mutations in PilA could change the packing of pilin subunits, allowing them to be disassembled more easily using only the torque provided by PilT. This hypothesis is supported by the location of the mutations at sites involved in inter-subunit interactions, based on cryoelectron microscopy reconstructions of assembled PAO1 pili (26, 67). In most suppressors we isolated, complementation with *pilU* further increased motility (Fig. 2B), as might be expected if mutant filaments were more easily disassembled compared with wild type, and PilU increased the available retraction force. In contrast, complementing the PilA suppressors with wild-type PilA caused concentration-dependent decreases in motility, suggesting that pili become increasingly more difficult to disassemble as the proportion of wild-type subunits increases. PilA mutations could also alter affinity for the platform protein PilC, allowing for more rapid transition of subunits between the pilus and inner membrane, or modulate interactions with other components such as PilNO that gate access of pilins to the assembly system (68). There are exceptions—the P22L and G54R pilins restored only limited motility in the *pilU* background, which decreased when *pilU* was expressed in *trans*. This phenotype is consistent with poor assembly that is further exacerbated when PilU increases disassembly rates.

Interestingly, the pilin mutations we identified are similar to those reported in a recent study examining the PilTU-independent retraction of *V. cholerae* Com pilins (PilA_com_), including G34, which is the equivalent of our promiscuous residue, A34 (Fig. S4) (47). In *V. cholerae*, DNA uptake requires pilus retraction. The authors noted residual levels of transformation (~4 logs lower than wild type) in the absence of both PilT and PilU, leading them to speculate that Com pilins might spontaneously disassemble. After ruling out involvement of the assembly ATPase PilB, they screened for mutants with increased transformation in the *pilTU* background, identifying four point mutations in PilA_com_ that mapped to the α1-C region of the N-terminal helix (G34) or the α-β loop (V74, G78, and G80). Each of these positions is predicted to contribute to inter-subunit packing and could thus alter disassembly rates. The pili produced by three of the four mutant subunits were shorter than wild type (47), similar to the phenotype we inferred for some of our suppressors, e.g., V28F and P42L, where despite the expression of wild-type levels of pilins intracellularly, few extracellular pili could be recovered by shearing (Fig. 2B). However, unlike the PilA_com_ suppressors, our mutant pilins did not restore twitching in the absence of PilT (Fig. 5). While our mutant pilins might be capable of some spontaneous disassembly, twitching motility—where pili are anchored to an immovable object and under high tension—requires substantially more retraction force than DNA uptake, making PilT essential.

As both structural and regulatory elements, pilins can impact bacterial physiology (42). Their sequences must be coordinately optimized for multiple traits (69), and fixation of some mutations might require adaptation by other parts of the system. Pilins must interact with one another with sufficient affinity to assemble a pilus that can withstand the forces of retraction but not so tightly that they cannot be easily disassembled. Their role as regulatory ligands depends on the maintenance of specific sequence motifs that are recognized by relevant sensors in the inner membrane (14, 23, 54, 55, 70). Finally, their exposure on the cell surface makes them common receptors for phages, so modifications that provide escape from phage predation are likely to be under positive selection (71–73). Our data suggest that for some positions, such as A34, diverse side-chain substitutions in PilA are well tolerated in terms of stability, but both sequence and context are important for function. For example, A34E and A34R have opposite motility and piliation phenotypes in a background lacking PilU (sequence), while A34S but not A34T twitching is PilU-dependent (context; Fig. 4). These data are consistent with previous findings from saturating mutagenesis studies of the *Neisseria meningitidis* major pilin PilE (69), where each residue was substituted on average with six others. A significant proportion of the mutations had no effect or beneficial effects on piliation, adherence, and intercellular aggregation. Interestingly, the authors of that study identified point mutants in the α1-N region of PilE that resulted in numerous short pili that failed to support pilus-mediated aggregation, similar to the short-pilus phenotypes reported for some mutant *V. cholerae* PilA_com_ (47) and our PilA suppressors. These mutant pilins may form filaments that are more likely to disassemble before reaching wild-type lengths.

This is the first study to report PilY1 point mutations that restore twitching motility in the absence of PilU. However, the mechanism remains unclear because the exact role of PilY1 in motility is not well established. It is an essential component of the pilus priming subcomplex, and without it, no pili are assembled (16). Its impact here is unlikely to be due to increased priming, since surface piliation of the PilY1 suppressors is similar to that of the Δ*pilU* parent (Fig. 2B). PilY1 is located at the pilus tip with the minor pilins, where it has been proposed to act as both a non-specific and specific adhesin (18, 20, 43, 74). The S59G mutation is located in the vWA-like domain that is thought to unfold when shear force is applied (14, 15, 18), and S59 forms part of a metal ion-dependent adhesion site (MIDAS) motif that binds divalent cations such as Ca^2+^, Mn^2+^, or Mg^2+^ to modulate adhesion (75). The MIDAS consensus sequence is D-x-S-x-S, where x is any amino acid; in PilY1, the sequence is ^55^D**-**D-S**-**G-S^59^. MIDAS motifs can bind Ca^2+^, Mg^2+^, and Mn^2+^, and substitution of bulkier Ca^2+^ with smaller Mn^2+^ increases ligand binding affinity and adhesiveness. The S59G mutation is predicted to alter divalent cation binding, and a more drastic mutation of the MIDAS motif in the *Kingella kingae* PilY1 homolog, PilC1, to A-x-A-x-A resulted in loss of adhesion (76). In *Streptococcus sanguis*, the combination minor pilin/adhesin PilB contains a vWA-like module with a MIDAS motif that is essential for twitching motility (77). Further work will be needed to connect PilY1 conformations to its role in twitching motility.

In addition to their non-twitching phenotype, *pilU* mutants have unique characteristics that suggest connections between PilU function and regulatory outcomes. Early studies noted their unusual matte colony morphology that might result from increased EPS production (78, 79). More recent work noted the similarity of PA14 *pilU* mutants to surface-conditioned wild-type cells, with high levels of cAMP and increased propensity for attachment (25). Here, we showed that PAO1 *pilU* mutants also have significantly elevated levels of cAMP compared to wild type, suggesting that surface sensing is dysregulated in the absence of PilU. We did not measure cAMP accumulation following the transition of cells from liquid to surfaces, since our focus was on whether restoration of surface-associated twitching motility in the *pilU* background would reduce its cAMP levels. Our data showed no correlation between the amount of twitching and cAMP levels, with the caveat that, for technical reasons, cAMP measurements were done using cells grown on minimal media, where Pil-Chp signaling may differ compared to the 1% LB medium used to measure motility. The PilA A34 point mutants identified here that confer similar levels of twitching in the presence and absence of PilU (e.g., A34T and A34M) will be valuable tools to study the specific contribution of PilU to surface sensing and modulation of cAMP levels.

While the complete signaling pathway from pilus surface engagement and force sensing to increased cAMP production via the adenylate cyclase CyaB remains to be defined, it is proposed to involve monitoring of transient changes in pilin conformation or abundance by the methyl-accepting chemotaxis protein PilJ of the Pil-Chp system plus other components such as the PilSR two-component system, SadC/BifA, and PilT (14, 23, 66, 70, 80). PilY1 is also considered a key component in surface sensing, with previous studies linking it to upregulation of virulence factor expression (14). Our data emphasize the involvement of PilY1 in twitching motility in addition to its role in pilus assembly, with the remarkable finding that single-residue changes in this enormous protein (~125 kDa) in either the vWA-like domain (S59G) or the C-terminal β-propeller domain (G1120D) restored partial motility in the Δ*pilU* background. Together, our data suggest that the bacterial response to surfaces reflects a complex interaction of PilU function with specific alleles of PilY1 and PilA that together modulate pilus dynamics and function.

## MATERIALS AND METHODS

### Bacterial strains and plasmids

Bacterial strains and plasmids used for this project are summarized in Tables S2 and S3. Bacterial strains were grown at 37°C overnight in LB or LB 1.5% agar unless otherwise specified. Where required for selection of plasmids, gentamicin at 30 mg/L (for *P. aeruginosa*) or 15 mg/L (for *Escherichia coli*) was added. For strains carrying arabinose-inducible vectors, L-arabinose was added to media at concentrations from 0.02% to 0.2% (wt/vol). Plasmids were introduced into chemically competent *E. coli* DH5α by heat shock and *E. coli* SM10 and/or *P. aeruginosa* by electroporation.

Plasmid constructs were made using standard cloning techniques and the restriction enzymes listed in Table S4. Deletion constructs were generated by cloning 500 bp up- and downstream of the corresponding gene plus a complementary region of 20 bp between the up- and downstream fragments to allow for overlap extension PCR. The products were then ligated into pEX18Gm. Knock-in constructs were generated using the same technique, with the up- and downstream regions flanking the point mutation, and the point mutation introduced in the complementary regions of the primers. Deletion and knock-in mutation constructs were introduced into *E. coli* SM10 and conjugated into the PAO1 parent strain as previously described (81). The mutations were confirmed with PCR and sequencing (Mobix, McMaster Genomics Facility, Hamilton).

### PilU protein purification

The *pilU* gene was amplified by PCR and cloned into the pET28a vector, incorporating an N-terminal 6× His tag for purification. The recombinant plasmid was confirmed by DNA sequencing and transformed into *E. coli* BL21 (DE3) cells for protein expression. Cultures were grown in LB medium supplemented with 50 µg/mL kanamycin at 37°C until reaching an OD_600_ of 0.7, at which point the temperature was reduced to 16°C. Protein expression was induced with 0.5 mM isopropyl β-D-1-thiogalactopyranoside, and cells were harvested via centrifugation (4,000 × *g*, 15 min, Beckman Avanti J-E) 16 h post-induction. Pellets were stored at −20°C until use.

For purification, thawed cell pellets were resuspended in buffer A1 (50 mM Tris [pH 9.0], 200 mM NaCl) and lysed using an Avestin EmulsiFlex C3 homogenizer. The lysate was clarified by centrifugation (40,000 × *g*, 40 min) and loaded onto a HiTrap Chelating HP column (GE Healthcare) pre-equilibrated with buffer A1 and buffer A2 (buffer A1 + 300 mM imidazole). PilU was eluted using a 20 mL gradient of buffer A2, with 25 µM ATP added to protein-containing fractions before concentration by centrifugation (4,000 × *g*). The sample was further concentrated using a 30K Amicon Ultra-15 spin concentrator and subjected to size-exclusion chromatography on a HiLoad 200 Superdex gel filtration column to separate PilU monomers from hexamers. SDS-PAGE confirmed protein purity, and the eluted protein was concentrated to 9 mg/mL using a 100K Amicon Ultra-15 spin concentrator. To optimize stability, purified PilU was screened under various buffer conditions containing glycerol, NaCl, and HEPES at different pH values. Solution light scattering from 20° to 70°C (Avacta Analytical UNit Optim 2) determined the aggregation temperature, leading to the identification of an optimized buffer [150 mM NaCl, 50 mM HEPES (pH 7.0), 250 mM (NH_4_)_2_SO_4_, 40 mM citric acid, and 20% glycerol], which replaced buffer A1 for subsequent experiments.

### PilU crystallization and structure determination

To enhance stability, 25 µM ATP was added to purified PilU prior to crystallization. The final buffer for crystallization contained 75 mM NaCl, 25 mM HEPES (pH 7.0), 125 mM (NH_4_)_2_SO_4_, 20 mM citric acid, 10% glycerol, 12.5 µM ATP, 11.5% PEG 8000, 50 mM Tris (pH 9.5), and 0.4 M LiCl. Initial screening was performed using the sitting-drop vapor diffusion method at 20°C with Top96 and MCSG 1 (Anatrace) crystallization screens. Promising conditions were refined in secondary optimization screens, leading to crystal formation in a reservoir solution containing 23% PEG 8000, 100 mM Tris (pH 9.5), and 0.8 M LiCl. Crystals were cryoprotected in 15% xylitol dissolved in the reservoir solution prior to vitrification in liquid nitrogen. Data to 4.5 Å resolution were collected at the National Synchrotron Light Source II and the structure solved using molecular replacement with subunits from the *C*6_CCCCCC_ conformation of PilT (35) (PDB 3JVV) and refined using PHENIX.refine (82). Four protomers were found in the asymmetric unit (Fig. S1). No density consistent with nucleotide was identified, nor was there density of sufficient quality to enable the modeling of the 30 C-terminal residues of PilU. As a consequence of the crystal packing, two nearly identical PilU hexamers with *C*3 symmetry could be identified. The data collection and refinement statistics are summarized in Table S1.

### Phylogenetic analysis of PilU and PilT homologs

To analyze evolutionary relationships, PilU and PilT orthologs were identified using HMMER (rp55 database) (83). Sequences were aligned using Clustal Omega, and a neighbor-joining phylogenetic tree was constructed in MEGA (84) with the Poisson substitution model. Sequence logos were generated using WebLogo (85) to visualize conserved residues.

### Twitching motility assays

Twitching motility assays were performed as described previously (40). Briefly, single colonies were stab-inoculated with a micropipette tip in 1% LB agar to the agar-plastic interface of cell culture-treated plates (OmniTray Cell Culture Treated, Thermo Fisher Scientific). Plates were incubated for 18 h at 37°C. Following incubation, the agar was carefully removed, and the plates were stained with 1% crystal violet for 10–15 min before washing with dH_2_O. Plates were imaged using a flatbed scanner, and twitching zones were measured using ImageJ (http://imagej.nih.gov/ij/; NIH, Bethesda, MD). Each strain was tested in biological duplicate with three technical replicates each.

### Ethyl methane sulfonate mutagenesis experiments

Gain-of-function mutants were generated using a modified chemical mutagenesis protocol (86–88). Briefly, *P. aeruginosa* PAO1 *pilU* was grown in 5 mL LB at 37°C shaking at 200 rpm overnight. One hundred microliters of the overnight culture was used to inoculate 5 mL LB, and strains were grown to an OD_600_ between 0.4 and 0.6. EMS was then added to the cultures to a final concentration of 5 µM, and the cultures were vortexed for 30 s before being incubated at 37°C without shaking for 1 h. Cells from 1 mL of culture were harvested by centrifugation for 1 min, and the supernatant was discarded. The cell pellet was washed twice in 1 mL 10 mM KPO_4_ (pH 7), then resuspended in 1 mL LB. Ten-microliter aliquots were spotted on LB 1.5% agar plates. The plates were incubated at room temperature for 5 days. Motile flares that emerged from the inoculum were picked and stab-inoculated into a 1% LB agar twitching assay plate that was incubated at 37°C overnight. After the agar was removed, twitching cells were scraped from the bottom of the plate and streaked for single colonies on LB 1.5% agar. Single colonies were assessed for twitching a second time before preparation of LB 15% glycerol stocks at –80°C. Whole genomic DNA (Wizard Genomic DNA Purification Kit) from gain-of-twitching isolates was subjected to whole-genome sequencing at Seqcenter (Pittsburgh, PA), then compared to the parent strain using breseq (41) to identify relevant mutations.

### Sheared surface protein assays

Sheared surface protein assays were done as described previously (40). Strains were grown from −80°C freezer stocks on 1.5% LB agar at 37°C overnight before streaking in a 5 × 5 grid pattern on another 1.5% LB agar plate and incubating in the same conditions overnight. Cells were scraped from the plate with a sterile coverslip and resuspended in 3 mL 1× phosphate-buffered saline (pH 7.3), then vortexed for 15 s (×2) before transferring to Eppendorf tubes and centrifuging for 30 min at 24,600 × *g*. The supernatant was transferred to a new tube and incubated in 10% 1 M MgCl_2_ overnight at 4°C. The supernatant was then centrifuged at 24,600 × *g* for 30 min, the supernatant was discarded, the resulting pellet was re-centrifuged at 24,600 × *g* for 1 min, and the remaining supernatant was removed by pipetting. The pellet was resuspended in 50 µL 1× SDS loading buffer and boiled for 10 min. Surface shearing assay products were stored at −20°C before use.

### SDS-PAGE and western blotting

Surface-sheared protein assays or cell pellets from 1 mL of culture at OD_600_ 0.6 were resuspended in 50 µL of 1× SDS-PAGE sample buffer and boiled for 10 min. SDS-PAGE samples were centrifuged at 24,600 × *g* for 1 min before loading into a 15% acrylamide SDS-PAGE gel. Proteins were separated at 80 V for 1 h. Gels were stained with Coomassie brilliant blue 1.2 mg/mL for 1 h then destained overnight. Gels for western blots were transferred to a nitrocellulose membrane at 225 mA for 1 h. The membrane was blocked overnight with 1× PBS 5% skimmed milk at 4°C before incubating overnight with 1:5,000 dilution of rabbit polyclonal anti-PilA antisera in 1× PBS at room temperature, shaking at 200 rpm. Unbound primary antibody was washed off with 1× PBS for 10 min at 200 rpm (×4) before incubating with goat anti-rabbit alkaline phosphatase conjugated secondary antibody at a 1:2,500 dilution for 1 h. The blot was resolved with 0.25 g/mL nitroblue tetrazolium and 0.1% 5-bromo-4-chloro-3-indolyl phosphate in 1× PBS before imaging (89).

### cAMP measurements

The *PaQa* reporter was transformed into bacterial strains of interest and used to estimate cAMP levels as previously described (14, 25). Briefly, strains were grown on M8 medium before being plated on M8 agar plates and incubated for 5 h. Cells were then harvested, washed, and resuspended in 1× PBS, and fluorescence was measured using a plate reader. The fluorescence intensity was normalized by the OD_600_ of the suspension.

### Surface-associated phenotypes

Biofilm assays were performed as described previously (90). Briefly, strains were inoculated from −80°C stocks in 5 mL LB and grown overnight, then subcultured in 50% LB, 50% PBS to a final OD_600_ of 0.1. One hundred fifty microliters of subculture was added in triplicate to wells of a 96-well TSP plate (Nunc), with media-only controls in the outermost wells to avoid edge effects. After the addition of a 96-peg ImmunoTSP lid (Nunc), the plate was incubated for 18 h at 37°C with shaking at 200 rpm in a sealed, humidified container. The peg lid was removed and rinsed in PBS to remove non-adherent cells, then stained for 15 min with 0.1% (wt/vol) crystal violet dye. After rinsing the lid with dH_2_O, bound dye was eluted into 150 µL of 33.3% acetic acid for 5 min and measured at 600 nm using a plate reader.

For swarming assays, we used DmGCA, which consists of Davis minimal medium (Sigma) containing 0.2% (wt/vol) glucose and 0.1% (wt/vol) casamino acids, solidified with 0.5% (wt/vol) agarose (Bioshop). This medium was poured into small 5 cm petri dishes and air-dried in a biosafety cabinet following solidification. Strains of interest were inoculated from single colonies from an LB agar plate to the center of the DmGCA plate and incubated at 37°C for 6 h before imaging on a flatbed scanner.

## SUPPLEMENTAL MATERIAL

Supplemental Figures mbio.02521-25-s0001.pdf Figures S1-S4.

Supplemental Tables mbio.02521-25-s0002.pdf Tables S1-S4.

ASM does not own the copyrights to Supplemental Material that may be linked to, or accessed through, an article. The authors have granted ASM a non-exclusive, world-wide license to publish the Supplemental Material files. Please contact the corresponding author directly for reuse.
