# Supplementary material for: Separation of Pseudomonas aeruginosa type IV pilus-dependent twitching motility and surface-sensing responses
Source: mBio. 2025 Oct 7;16(11):e02521-25. doi: 10.1128/mbio.02521-25 (PMC12607626; doi:10.1128/mbio.02521-25)
Supplement: Supplemental Figures — Figures S1-S4. [file mbio.02521-25-s0001.pdf]

Supplementary Figures S1-S4 for: Separation of *Pseudomonas aeruginosa* type IV pilus-dependent twitching motility and surface-sensing responses

Rebecca Barnshaw[1#], Hanjeong Harvey[1#], Matthew McCallum[2,3,$], Tomas Lazarou[3,$], Sheryl Nguyen[3], Ikram Qaderi[1], Veronica Tran[1], Nathan Roberge[1], Christopher Geiger[4], George A. O'Toole[4], P. Lynne Howell[3] and Lori L. Burrows[1]*. [#]these authors contributed equally

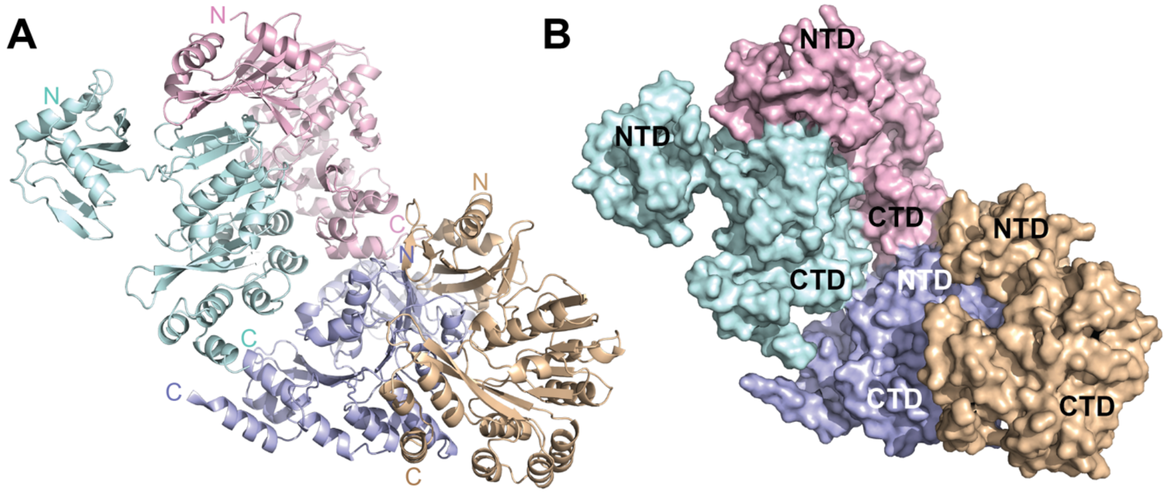

Supplementary Figure S1. The asymmetric unit of the PilU X-ray crystal structure contains 4 promoters. Four monomers of PilU were found in the asymmetric unit, shown as **A.** individually colored ribbon or **B.** space-filling models, with N-terminal (NTD) and C-terminal (CTD) domains labelled. The N-terminal domain of one monomer packs onto the C-terminal domain of the adjacent subunit.

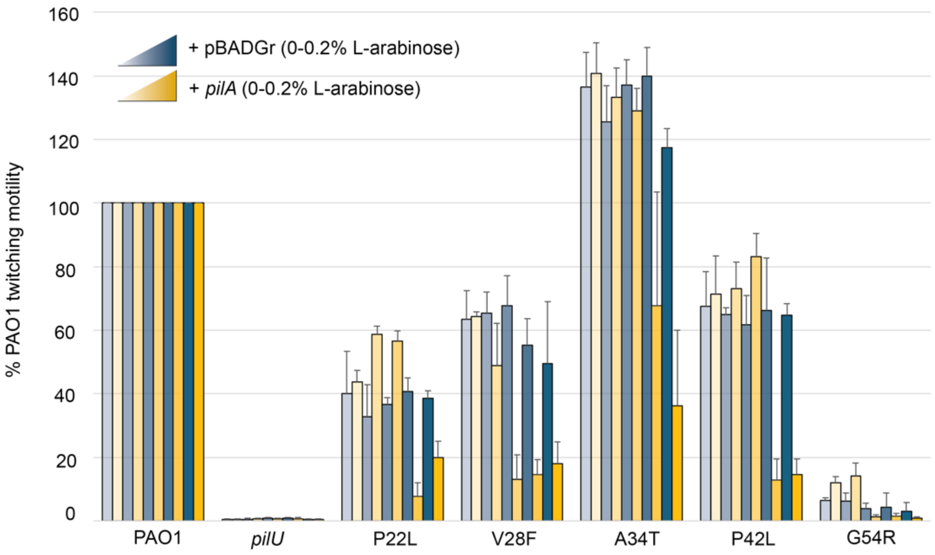

Supplementary Figure S2. Quantification of twitching for a representative subset of PilA suppressors complemented with wild-type PilA. Suppressor mutants with chromosomal point mutations in PilA were complemented with empty vector (pBADGr, blue bars) or pBADGr encoding wild-type PilA (yellow bars), and PilA expression was induced with increasing amounts of L-arabinose, from 0, 0.02, 0.05, 0.1 to 0.2% (w/v), shown with lighter to darker shading. Increasing expression of wild-type pilins (yellow) led to a dose-dependent decrease in twitching in the suppressor mutants.

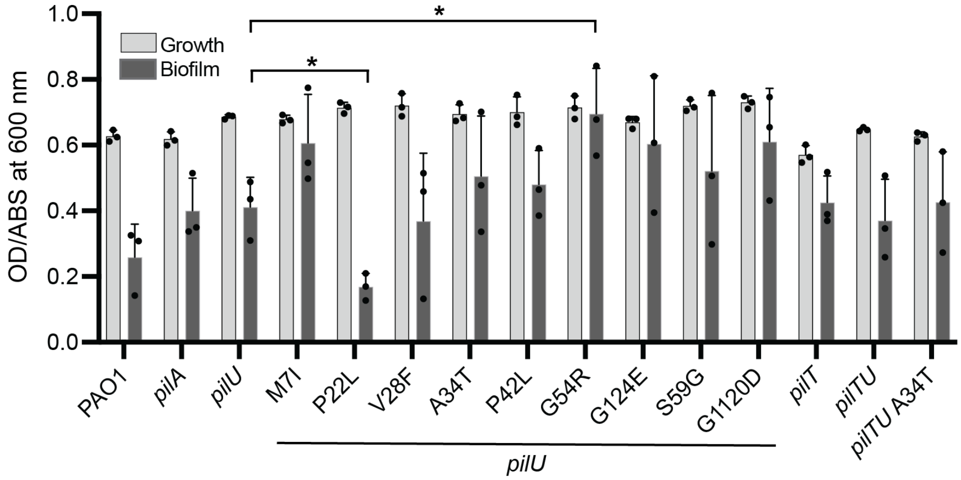

Supplementary Figure S3. Biofilm formation by *pilU* suppressor mutants. Biofilm formation was measured using 96 well peg-lid assays. The growth of each strain (OD_{600}, light grey bars) and the amount of biofilm formed on the pegs (dark grey bars) as measured by the amount of bound crystal violet dye (ABS_{600}) was measured. While there were no differences in growth between strains, the P22L and G54R mutants made significantly less and more biofilm, respectively, compared to their *pilU* parent. Each point represents the average of technical triplicates and each bar represents the average of 3 biological replicates.

```
      1     7     11        21 22     28   31  34     41 42
PAO1  FTLIELMIVV AIIGILAAIA IPQYQNYVAR SEGASALATI NPLKTTVEES
Com   FTLIELMIVV AVIGVLAAIA IPQYQNYVKK SAIGVGLANI TALKTNIEDY
                                          34

      51  54     61         70         80         90
PAO1  LSRGIAGSKI KIGTTASTA- TETYVGVEPD ANKLGVIAVA IEDSGA—GDI
Com   IATE--GS—F PATTAGTAAG FTRLGTVEDM GD-GKIVIAP TASGALGGTI
                                   74  78  80

      99     124 109        119        129        139
PAO1  TFTFQTGTSS PKNATKVITL NRTADGVWAC KSTQDPMFTP KGCD-----N
Com   KYTFDAGVVS S----SKIQL ARDANGLWTC STTVTSEIAP KGCTAGATIN
```

Supplementary Figure S4. Comparison of suppressor mutations in *Pseudomonas aeruginosa* and *Vibrio cholerae* Com pilins. A previous study (Chlebek JL et al., PNAS 118:e2102780118, 2021) of ATPase-independent retraction of *V. cholerae* (Com) competence pili identifed 4 suppressor mutants in $PilA_{com}$ that led to increased rates of transformation in the absence of PilT and PilU. Alignment of the PAO1 and $PilA_{com}$ pilins using MUSCLE shows identical residues in black, residues that differ in $PilA_{com}$ versus PilA in blue, and the relative positions of the point mutations identified in this study (green) and in $PilA_{com}$ (purple), including the common A34/G34 site. Unlike in $PilA_{com}$, restoration of pilus function in *P. aeruginosa* was dependent on PilT.
