# Supplementary material for: Separation of Pseudomonas aeruginosa type IV pilus-dependent twitching motility and surface-sensing responses
Source: mBio. 2025 Oct 7;16(11):e02521-25. doi: 10.1128/mbio.02521-25 (PMC12607626; doi:10.1128/mbio.02521-25)
Supplement: Supplemental Tables — Tables S1-S4. [file mbio.02521-25-s0002.pdf]

Supplementary Tables S1-S4 for: Separation of *Pseudomonas aeruginosa* type IV pilus-dependent twitching motility and surface-sensing responses

Rebecca Barnshaw[1#], Hanjeong Harvey[1#], Matthew McCallum[2,3,$], Tomas Lazarou[3,$], Sheryl Nguyen[3], Ikram Qaderi[1], Veronica Tran[1], Nathan Roberge[1], Christopher Geiger[4], George A. O'Toole[4], P. Lynne Howell[3] and Lori L. Burrows[1*]. [#]these authors contributed equally

Supplementary Table S1. PilU Crystal Structure Data Collection and Refinement Statistics

**Crystal structure name**	**PilU**
**Data collection**	
Facility	NSLS-II
Beamline	17-ID-1
Wavelength (Å)	0.999614
Space group	$P2_13$
a, b, c (Å)	191.0, 191.0, 191.0
α, β, γ (°)	90, 90, 90
Resolution (Å)	30-4.5<br>(4.7-4.5)
Total Reflections	265669
Unique Reflections	25631 (2584)
Redundancy	10.4 (8.5)
Completeness (%)	99 (87)
Mean I / σI	5.9 (1.2)
R_{pim} (%)**	6.2 (44)
CC* (%)**	100 (82)
**Refinement**	
R_{work} / R_{free} (%)†	28.1 / 29.5
RMSD<br>Bond lengths (Å)<br>Bond angles (°)<br>Ramachandran‡<br>Favoured (%)<br>Allowed (%)<br>Outliers (%)	 0.001<br>0.39<br><br>98<br>100<br>0
Coordinate error (Å)§	0.62
Atoms	8507
Protein	8507
Av. B-factors (Å^2)	249
Protein	249
PDB ID	6N32

Note: Values in parentheses correspond to the highest resolution shell.

*, Atypical completeness of the CCOCCO PilT structures reflects anisotropic truncation

**, $R_{Sym} = \sum\sum | I - \langle I \rangle | / \sum\sum I$, $R_{Pim} = \sum\sqrt{1/(n-1)} \sum | I - \langle I \rangle | / \sum\sum I$, and $CC^* = \sqrt{(2CC_{1/2}/(1+CC_{1/2}))}$ where $CC_{1/2}$ is the Pearson correlation coefficient of two half data sets as described elsewhere (1).

†, $R_{work} = \sum | |F_{obs}| - k|F_{calc}| | / |F_{obs}|$ where F_{obs} and F_{calc} are the observed and calculated structure factors, respectively. R_{free} is the sum extended over a subset of reflections (5%) excluded from all stages of the refinement

§, Maximum-likelihood based Coordinate Error, as determined by *PHENIX* (2).

Supplementary Table S2. Bacterial strains used in this study

Strain	Characteristics	Source/Reference
***E. coli* strains**		
E. coli DH5α	F- *φ80lacZΔM15 Δ(lacZYA-argF)U169 recA1 endA1 hsdR17(rk-, mk+) phoA supE44 thi-1 gyrA96 relA1 λ*	Invitrogen
E. coli SM10	*thi-1 thr leu tonA lacY supE recA::RP4-2- Tc::Mu* (KmR)	(3)
***P. aeruginosa* strains**		
mPAO1 WT	WT	(4)
mPAO1 WT + pUCP20Gm	WT with pUCP20Gm	Burrows lab
mPAO1 WT + pUCP20Gm-*pilU*	WT with pUCP20Gm containing *pilU*	(This work)
mPAO1 WT + pBADGr	WT with pBADGr	Burrows lab
mPAO1 WT + pBADGr-*pilA*	WT with pBADGr containing *pilA*	Burrows lab
mPAO1 *ΔpilU*	Chromosomal deletion of *pilU*	(This work)
mPAO1 *ΔpilU* + pUCP20Gm	Chromosomal deletion of *pilU* with pUCP20Gm	(This work)
mPAO1 *ΔpilU* + pUCP20Gm-*pilU*	Chromosomal deletion of *pilU* with pUCP20Gm containing *pilU*	(This work)
mPAO1 *ΔpilU* + pBADGr	Chromosomal deletion of *pilU* with pBADGr	(This work)
mPAO1 *ΔpilU* + pBADGr-*pilA*	Chromosomal deletion of *pilU* with pBADGr containing *pilA*	(This work)
mPAO1 *ΔpilU* 2mut19c1	twitching suppressor of *pilU*	(This work)
mPAO1 *ΔpilU* 2mut22c1	twitching suppressor of *pilU*	(This work)
mPAO1 *ΔpilU* 2mut24c1	twitching suppressor of *pilU*	(This work)
mPAO1 *ΔpilU* 2mut31c2	twitching suppressor of *pilU*	(This work)
mPAO1 *ΔpilU* 2mut34c1	twitching suppressor of *pilU*	(This work)
mPAO1 *ΔpilU* 2mut48c2	twitching suppressor of *pilU*	(This work)
mPAO1 *ΔpilU* 2mut51c1	twitching suppressor of *pilU*	(This work)
mPAO1 *ΔpilU* 2mut53c1	twitching suppressor of *pilU*	(This work)
mPAO1 *ΔpilU* 2mut58c1	twitching suppressor of *pilU*	(This work)
mPAO1 *ΔpilU* 2mut58c2	twitching suppressor of *pilU*	(This work)
mPAO1 *ΔpilU* 2mut70c1	twitching suppressor of *pilU*	(This work)
mPAO1 *ΔpilU* 2mut71c1	twitching suppressor of *pilU*	(This work)

mPAO1 *ΔpilU* PilA M7I	Chromosomal deletion of *pilU* and a chromosomal substitution of PilA M7 to isoleucine	(This work)
mPAO1 *ΔpilU* PilA P22L	Chromosomal deletion of *pilU* and a chromosomal substitution of PilA P22 to leucine	(This work)
mPAO1 *ΔpilU* PilA V28F	Chromosomal deletion of *pilU* and a chromosomal substitution of PilA V28 to phenylalanine	(This work)
mPAO1 *ΔpilU* PilA A34T	Chromosomal deletion of *pilU* and a chromosomal substitution of PilA A34 to threonine	(This work)
mPAO1 *ΔpilU* PilA P42L	Chromosomal deletion of *pilU* and a chromosomal substitution of PilA P42 to leucine	(This work)
mPAO1 *ΔpilU* PilA G54R	Chromosomal deletion of *pilU* and a chromosomal substitution of PilA G54 to arginine	(This work)
mPAO1 *ΔpilU* PilA G124E	Chromosomal deletion of *pilU* and a chromosomal substitution of PilA G124 to glutamate	(This work)
mPAO1 *ΔpilU* PilA M7I + pUCP20Gm	Chromosomal deletion of *pilU* and a chromosomal substitution of PilA M7 to isoleucine with pUCP20Gm	(This work)
mPAO1 *ΔpilU* PilA P22L + pUCP20Gm	Chromosomal deletion of *pilU* and a chromosomal substitution of PilA P22 to leucine with pUCP20Gm	(This work)
mPAO1 *ΔpilU* PilA V28F + pUCP20Gm	Chromosomal deletion of *pilU* and a chromosomal substitution of PilA V28 to phenylalanine with pUCP20Gm	(This work)
mPAO1 *ΔpilU* PilA A34T + pUCP20Gm	Chromosomal deletion of *pilU* and a chromosomal substitution of PilA A34 to threonine with pUCP20Gm	(This work)
mPAO1 *ΔpilU* PilA P42L + pUCP20Gm	Chromosomal deletion of *pilU* and a chromosomal substitution of PilA P42 to leucine with pUCP20Gm	(This work)
mPAO1 *ΔpilU* PilA G54R + pUCP20Gm	Chromosomal deletion of *pilU* and a chromosomal substitution of PilA G54 to arginine with pUCP20Gm	(This work)
mPAO1 *ΔpilU* PilA G124E + pUCP20Gm	Chromosomal deletion of *pilU* and a chromosomal substitution of PilA G124 to glutamate with pUCP20Gm	(This work)
mPAO1 PilA *ΔpilU* M7I + pUCP20Gm-*pilU*	Chromosomal deletion of *pilU* and a chromosomal substitution of PilA M7 to isoleucine with pUCP20Gm containing *pilU*	(This work)

mPAO1 Δ*pilU* PilA P22L + pUCP20Gm-*pilU*	Chromosomal deletion of *pilU* and a chromosomal substitution of PilA P22 to leucine with pUCP20Gm containing *pilU*	(This work)
mPAO1 Δ*pilU* PilA V28F + pUCP20Gm-*pilU*	Chromosomal deletion of *pilU* and a chromosomal substitution of PilA V28 to phenylalanine with pUCP20Gm containing *pilU*	(This work)
mPAO1 Δ*pilU* PilA A34T + pUCP20Gm-*pilU*	Chromosomal deletion of *pilU* and a chromosomal substitution of PilA A34 to threonine with pUCP20Gm containing *pilU*	(This work)
mPAO1 Δ*pilU* PilA P42L + pUCP20Gm-*pilU*	Chromosomal deletion of *pilU* and a chromosomal substitution of PilA P42 to leucine with pUCP20Gm containing *pilU*	(This work)
mPAO1 Δ*pilU* PilA G54R + pUCP20Gm-*pilU*	Chromosomal deletion of *pilU* and a chromosomal substitution of PilA G54 to arginine with pUCP20Gm containing *pilU*	(This work)
mPAO1 Δ*pilU* PilA G124E + pUCP20Gm-*pilU*	Chromosomal deletion of *pilU* and a chromosomal substitution of PilA G124 to glutamate with pUCP20Gm containing *pilU*	(This work)
mPAO1 Δ*pilU* PilA M7I + pBADGr	Chromosomal deletion of *pilU* and a chromosomal substitution of PilA M7 to isoleucine with pBADGr	(This work)
mPAO1 Δ*pilU* PilA P22L + pBADGr	Chromosomal deletion of *pilU* and a chromosomal substitution of PilA P22 to leucine with pBADGr	(This work)
mPAO1 Δ*pilU* PilA V28F + pBADGr	Chromosomal deletion of *pilU* and a chromosomal substitution of PilA V28 to phenylalanine with pBADGr	(This work)
mPAO1 Δ*pilU* PilA A34T + pBADGr	Chromosomal deletion of *pilU* and a chromosomal substitution of PilA A34 to threonine with pBADGr	(This work)
mPAO1 Δ*pilU* PilA P42L + pBADGr	Chromosomal deletion of *pilU* and a chromosomal substitution of PilA P42 to leucine with pBADGr	(This work)
mPAO1 Δ*pilU* PilA G54R + pBADGr	Chromosomal deletion of *pilU* and a chromosomal substitution of PilA G54 to arginine with pBADGr	(This work)
mPAO1 Δ*pilU* PilA G124E + pBADGr	Chromosomal deletion of *pilU* and a chromosomal substitution of PilA G124 to glutamate with pBADGr	(This work)
mPAO1 Δ*pilU* PilA M7I + pBADGr-*pilA*	Chromosomal deletion of *pilU* and a chromosomal substitution of PilA M7	(This work)

	to isoleucine with pBADGr containing *pilA*	
mPAO1 *ΔpilU* PilA P22L + pBADGr-*pilA*	Chromosomal deletion of *pilU* and a chromosomal substitution of PilA P22 to leucine with pBADGr containing *pilA*	(This work)
mPAO1 *ΔpilU* PilA V28F + pBADGr-*pilA*	Chromosomal deletion of *pilU* and a chromosomal substitution of PilA V28 to phenylalanine with pBADGr containing *pilA*	(This work)
mPAO1 *ΔpilU* PilA A34T + pBADGr-*pilA*	Chromosomal deletion of *pilU* and a chromosomal substitution of PilA A34 to threonine with pBADGr containing *pilA*	(This work)
mPAO1 *ΔpilU* PilA P42L + pBADGr-*pilA*	Chromosomal deletion of *pilU* and a chromosomal substitution of PilA P42 to leucine with pBADGr containing *pilA*	(This work)
mPAO1 *ΔpilU* PilA G54R + pBADGr-*pilA*	Chromosomal deletion of *pilU* and a chromosomal substitution of PilA G54 to arginine with pBADGr containing *pilA*	(This work)
mPAO1 *ΔpilU* PilA G124E + pBADGr-*pilA*	Chromosomal deletion of *pilU* and a chromosomal substitution of PilA G124 to glutamate with pBADGr containing *pilA*	(This work)
mPAO1 *ΔfliC*	Chromosomal deletion of *fliC*	Burrows lab
mPAO1 *ΔpilU ΔfliC*	Chromosomal deletion of *pilU* and *fliC*	(This work)
mPAO1 *ΔpilT ΔfliC*	Chromsomal deletion of *pilT* and *fliC*	(This work)
mPAO1 *ΔpilT*	Chromsomal deletion of *pilT*	(This work)
mPAO1 *ΔpilT ΔpilU*	Chromsomal deletion of *pilT* and *pilU*	(This work)
mPAO1 *ΔpilT ΔpilU* PilA A34T	Chromsomal deletion of *pilT*, *pilU* and a chromosomal substitution of PilA α-helical residue A34 to threonine	(This work)
mPAO1 *ΔpilU PilY1* S59G	Chromosomal deletion of *pilU* and a chromosomal substitution of PilY1 residue S59 to glycine	(This work)
mPAO1 *ΔpilU* PilY1 G1120E	Chromosomal deletion of *pilU* and a chromosomal substitution of PilY1 residue G1120 to glutamate	(This work)
mPAO1 *ΔsadC* + pBADGr-*sadC*	Chromosomal deletion of *sadC* with pBADGr containing *sadC*	(5)

Supplementary Table S3. Plasmids used in this study

Vector	Characteristics	Source
pEX18Gm	Suicide vector used for gene replacement	(6)
pUCP20Gm	Shuttle vector with SmaI-flanked Gm cassette inserted into ScaI site in *bla*	(7)
pBADGr	Broad host range arabinose inducible vector used for complementation; ori araC-PBAD Gmr mob+	(8)
pEX18Gm-*ΔpilU*	pEX18Gm with a *pilU* deletion construct	(This work)
pEX18Gm-*ΔpilT*	pEX18Gm with a *pilT* deletion construct	Burrows lab
pEX18Gm-*ΔfliC*	pEX18Gm with a *fliC* deletion construct	Burrows lab
pEX18Gm-*ΔpilT ΔpilU*	pEX18Gm with a *pilT* deletion construct for use in a *ΔpilU* background	(This work)
pEX18Gm-*pilA* M7I	pEX18Gm with a knock-in construct for a substitution of PilA M7 to isoleucine	(This work)
pEX18Gm-*pilA* P22L	pEX18Gm with a knock-in construct for a substitution of PilA P22 to leucine	(This work)
pEX18Gm-*pilA* V28F	pEX18Gm with a knockin construct for a substitution of PilA V28 to phenylalanine	(This work)
pEX18Gm-*pilA* A34T	pEX18Gm with a knockin construct for a substitution of PilA A34 to threonine	(This work)
pEX18Gm-*pilA* P42L	pEX18Gm with a knockin construct for a substitution of PilA P42 to leucine	(This work)
pEX18Gm-*pilA* G54R	pEX18Gm with a knockin construct for a substitution of PilA G54 to arginine	(This work)
pEX18Gm-*pilA* G124E	pEX18Gm with a knockin construct for a substitution of PilA G124 to glutamate	(This work)
pUCP20Gm-*pilU*	pUCP20Gm expressing *pilU*	(This work)
pBADGr-*pilA*	pBADGr expressing *pilA*	Burrows lab
pBADGr-*sadC*	pBADGr expressing *sadC*	(5)

Supplementary Table S4. Primers used in this study [a,b].

Primer Name	Sequence (5'→3')
pilU F	ATTAGGTACCATCATGGAATTCGAAAAGC
pilU R	ATTACTGCAGTCAGCGGAAGCGCCGGCCG
pilA seq F	CCCTCTGAACGAATCGCAGG
pilA seq R	GCTGCCAAATCGAGGAAATCC
pilU F1	ATTAGAGCTCAATCGGTGATCTCGCAGACC
pilU F2	AACTCGAGCCGCAAGCATGCTGAACTGAGCCTGGAAATCACCGA
pilU R1	TTCAGCATGCTTGCGGCTCGAGTTGGAGCCGCCCTTTTCCAC
pilU R2	ATTAAAGCTTGCAACAGCCTGAACGTCAAG
pilC F1	ATTCGAGCTCATCCGCCAGGTACGCAATGG
pilC R1	GGTCGGTACCCTTCGTTCTGCGAGTCTTCC
pilC F2	CGCTGGTACCCTACATCCAGGGCTACTACC
pilC R2	GGATAAGCTTCGCCTGGGTTACAAGACCGC
pilTpilU F1	ATTAGAGCTCGTTCAAGGGCAGGTCGGCCA

pilTpilU R1	GGGCGTTCTCGCGGCTGATGGTCCGAAGCGCCCTGTTTG
pilTpilU F2	CAAACAGGGCGCTTCGGACCATCAGCCGCGAGAACGCCC
pilTpilU R2	ATTAAAGCTTGGTCGATCACCAGCGGCAAG
pilA M7I F1	ATTAGAGCTCCCATTAGAGGAACCCAATCA
pilA M7I R1	CGAACTGAT**A**ATCGTGGTTG
pilA M7I F2	CAACCACGAT**T**ATCAGTTCG
pilA M7I/P22L R2	ATTAAAGCTTGCTATTCAGGTCGCAATAGG
pilA P22L/V28F F1	ATTAGAGCTCCCTCTGAACGAATCGCAGG
pilA P22L R1	ATTGCCATTC**T**CCAGTATCAG
pilA P22L F2	CTGATACTGG**A**GAATGGCAAT
pilA V28F R1	CAGAACTAT**T**TTGCGCGTTCG
pilA V28F F2	CGAACGCGCAA**A**ATAGTTCTG
pilA V28F R2	ATTAAAGCTTGCTATTCAGGTCGCAATAGGC
pilA A34T F1	ATTAGAGCTCCATGCGCCTCACCCTCTGA
pilA A34T R1	GTTCGGAAGGT**A**CTTCGGCG
pilA A34T F2	CGCCGAAG**T**ACCTTCCGAAC
pilA A34T R2	ATTAAAGCTTCGAACTGCTCGGCGGACA
pilA P42L/G54R F1	ATTAGAGCTCCTACATCTCCATCGGCACC
pilA P42L R1	CGATCAACC**T**GCTGAAGACC
pilA P42L F2	GGTCTTCAGC**A**GGTTGATCG
pilA P42L/G54R R2	ATTAAAGCTTCACCAGCGACAGCTTGTTG
pilA G54R R1	GCTGTCGCGT**A**GAATTGCTG
pilA G54R F2	CAGCAATTC**T**ACGCGACAGC
pilA G124E F1	ATTAGAGCTCGCATAGCACCCGGCAAGCC
pilA G124E R1	TGCGGATG**A**GGTCTGGGCTT
pilA G124E F2	AAGCCCAGACC**T**CATCCGCA
pilA G124E R2	ATTAAAGCTTGGACAGGCCGCTCAGTTGG

[a]-Restriction sites are underlined

[b]-Mismatched bases for site directed mutagenesis are bolded

Supplementary References:

1. Karplus PA, Diederichs K. 2012. Linking crystallographic model and data quality. Science 336:1030-3.
2. Adams PD, Afonine PV, Bunkoczi G, Chen VB, Davis IW, Echols N, Headd JJ, Hung LW, Kapral GJ, Grosse-Kunstleve RW, McCoy AJ, Moriarty NW, Oeffner R, Read RJ, Richardson DC, Richardson JS, Terwilliger TC, Zwart PH. 2010. PHENIX: a comprehensive Python-based system for macromolecular structure solution. Acta Crystallogr D Biol Crystallogr 66:213-21.
3. Simon R, Priefer U, Pühler A. 1983. A broad host range mobilization system for in vivo genetic engineering: transposon mutagenesis in gram negative bacteria. Bio/technology 1:784-791.
4. Jacobs MA, Alwood A, Thaipisuttikul I, Spencer D, Haugen E, Ernst S, Will O, Kaul R, Raymond C, Levy R. 2003. Comprehensive transposon mutant library of *Pseudomonas aeruginosa*. Proceedings of the National Academy of Sciences 100:14339-14344.

5. Marko VA, Kilmury SL, MacNeil LT, Burrows LL. 2018. *Pseudomonas aeruginosa* type IV minor pilins and PilY1 regulate virulence by modulating FimS-AlgR activity. PLoS Pathogens 14:e1007074.
6. Hoang TT, Karkhoff-Schweizer RR, Kutchma AJ, Schweizer HP. 1998. A broad-host-range Flp-FRT recombination system for site-specific excision of chromosomally-located DNA sequences: application for isolation of unmarked *Pseudomonas aeruginosa* mutants. Gene 212:77-86.
7. Chiang P, Burrows LL. 2003. Biofilm formation by hyperpiliated mutants of *Pseudomonas aeruginosa*. Journal of Bacteriology 185:2374-2378.
8. Giltner CL, Habash M, Burrows LL. 2010. *Pseudomonas aeruginosa* minor pilins are incorporated into type IV pili. Journal of Molecular Biology 398:444-461.
